# Cripto-1 as a Key Factor in Tumor Progression, Epithelial to Mesenchymal Transition and Cancer Stem Cells

**DOI:** 10.3390/ijms22179280

**Published:** 2021-08-27

**Authors:** Hilal Arnouk, Gloria Yum, Dean Shah

**Affiliations:** 1 Department of Pathology, College of Graduate Studies, Midwestern University, Downers Grove, IL 60515, USA; 2 Chicago College of Optometry, Midwestern University, Downers Grove, IL 60515, USA; gloria.yum@midwestern.edu; 3 Chicago College of Osteopathic Medicine, Midwestern University, Downers Grove, IL 60515, USA; dean.shah@midwestern.edu; 4 College of Dental Medicine-Illinois, Midwestern University, Downers Grove, IL 60515, USA; 5 Master of Public Health Program, College of Graduate Studies, Midwestern University, Downers Grove, IL 60515, USA

**Keywords:** Cripto-1, tumor progression, EMT, epithelial to mesenchymal transition, cancer stem cells, biomarker, therapeutic target, breast cancer, melanoma, metastasis

## Abstract

Cripto-1 is an essential protein for human development that plays a key role in the early phase of gastrulation in the differentiation of an embryo as well as assists with wound healing processes. Importantly, Cripto-1 induces epithelial to mesenchymal transition to turn fixed epithelial cells into a more mobile mesenchymal phenotype through the downregulation of epithelial adhesion molecules such as E-cadherin, occludins, and claudins, and the upregulation of mesenchymal, mobile proteins, such as N-cadherin, Snail, and Slug. Consequently, Cripto-1’s role in inducing EMT to promote cell motility is beneficial in embryogenesis, but detrimental in the formation, progression and metastasis of malignant tumors. Indeed, Cripto-1 is found to be upregulated in most cancers, such as breast, lung, gastrointestinal, hepatic, renal, cervical, ovarian, prostate, and skin cancers. Through its role in EMT, Cripto-1 can remodel cancer cells to enable them to travel through the extracellular matrix as well as blood and lymphatic vessels to metastasize to different organs. Additionally, Cripto-1 promotes the survival of cancer stem cells, which can lead to relapse in cancer patients.

## 1. Cripto-1: Role in Cell Biology

**Figure 1:**
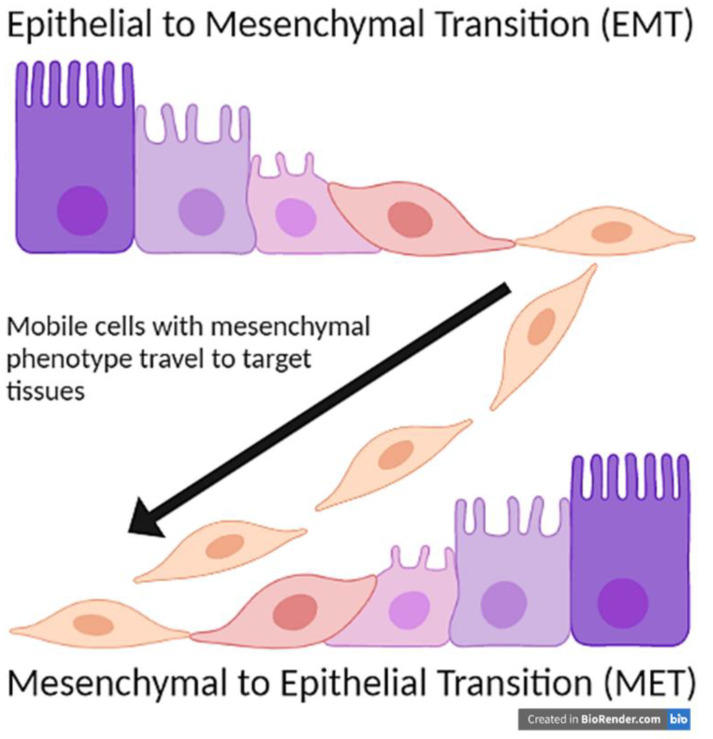
Epithelial to Mesenchymal Transition (EMT) and Mesenchymal to Epithelial Transition (MET).

Cripto-1 is a protein encoded by the teratocarcinoma-derived growth factor-1 (TDGF-1) gene [1]. It is a protein that is found to play a role in embryogenesis, cell migration, and tumor progression through a process called epithelial to mesenchymal transition (EMT) where fixed epithelial cells revert to mobile mesenchymal phenotype and migrate to other areas of the body (Figure 1). Thus, Cripto-1 is necessary during embryonic development. However, it has a pathological role in promoting tumor progression and metastasis.

Cripto-1 is also influenced by other signaling molecules, specifically Wnt and β-catenin [2]. These proteins trigger cardiomyocyte differentiation [3], as well as mesoderm patterning and cardiac specification [4]. Mutations in TDGF-1 are associated with the development of ventricular septal defect, the most common congenital heart defect [1]. 

Cripto-1 is also the co-receptor for Nodal, a protein in the transforming growth factor-β (TGF-β) family. TGF-β proteins play a role in differentiation and stem cell regulation. Nodal assists with gastrulation, organ positioning, and symmetry [5]. Both Nodal and Cripto-1 are needed for full functionality [6]. This article will focus on the role of Cripto-1 in EMT and cancer.

## 2. Epithelial to Mesenchymal Transition (EMT)

**Figure 2:**
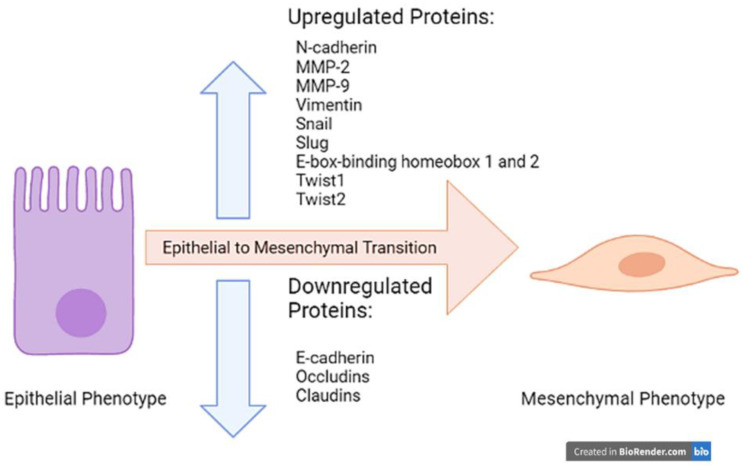
Upregulated and Downregulated Proteins in EMT.

An important function of Cripto-1 is to induce epithelial to mesenchymal transition (EMT) during embryogenesis [7,8,9,10,11,12,13,14]. EMT primarily functions to mobilize cells to different parts of the body through a two-part mechanism. First, there is a downregulation of the epithelial adherence proteins. Loss of these proteins causes the cells to no longer be tightly held together, thus giving them the opportunity to move and migrate (Figure 1). Examples of these proteins that are downregulated in EMT include E-cadherin and tight junction proteins, such as occludins and claudins (Figure 2) [15,16,17].

E-cadherin is crucial to cell-cell adhesion, particularly in epithelial tissues [18]. It uses calcium to link protein dimers between two adjacent cells, and the cadherin protein dimers are anchored to the cytoplasmic side of the plasma membrane through binding with catenin proteins [18]. Downregulation of E-cadherin is a necessary step in EMT, allowing the cells to become more mobile and to migrate to their intended destination. Occludins and claudins form tight junctions between cells and, similar to E-cadherin, have intracellular, transmembrane, and extracellular components by which they attach [19]. While occludins are not necessary for tight junction formation alone, the loss of these proteins can be detrimental to maintaining the architecture of epithelial tissues. Additionally, knockout studies in mice have shown that the absence of different forms of claudin disrupts epidermal tissue and blood-brain barrier functions [19], which leads to leakage between adjacent cells and enables individual cells to be more mobile. The activation of matrix metalloproteinases 2 and 9 (MMP-2 and MMP-9) also aid in the loss of the epithelial phenotype (Figure 2). Matrix metalloproteinases degrade the basal lamina, specifically by cleaving type IV collagen [20]. Thus, MMP-2 and MMP-9 remove the anchor of the cell to the epithelial lining. 

The second step in the EMT mechanism is the upregulation of mesenchymal proteins. Most notably, N-cadherin replaces the lost adherence molecules such as E-cadherin (Figure 1) [21]. N-cadherin does not play a role in adherence, so the cell will become more mobile, a characteristic of mesenchymal cells. Unlike E-cadherin, N-cadherin promotes the aggregation of cells and invasion of malignant cells [22,23]. N-cadherin also promotes survival in certain cancer cells, particularly in melanoma and prostate cancer cell lines [24]. It recruits PI3K, which activates Akt. Akt, in turn, inactivates the pro-apoptotic molecule, Bad. Thus, N-cadherin inhibits apoptosis in these cancer cells [24]. N-cadherin plays a role in angiogenesis and neuroectoderm vascularization as well, which is prevalent in non-small cell lung cancer [24]. A natural downregulation of N-cadherin in endothelial cells in embryonic development forms the blood-retina and blood-brain barriers [25]. 

Other proteins of the mesenchymal phenotype, such as Vimentin, are upregulated as well in EMT (Figure 2) [7]. In addition, there is an increase in certain transcription factors such as the zinc finger proteins Snail, Slug, E-box-binding homeobox 1 and 2 and helix-loop-helix factors Twist1 and Twist2 (Figure 2) [26]. These proteins bind DNA and modify transcription to form a more mesenchymal phenotype. The resulting change in epithelial proteins to their mesenchymal counterparts that usually accompanies EMT gives rise to leaky membranes, which can be detrimental to certain barriers such as the blood-brain barrier. 

Epithelial to mesenchymal transition primarily plays a physiological role in gastrulation. Ectodermal cells leave the surface, undergo EMT, and then travel to form all three cell layers [27]. This process is required for the formation of the paraxial, intermediate, and lateral plate mesoderm layers, which are responsible for the development of the musculoskeletal, urogenital, and gastrointestinal systems respectively [28]. However, for the newly-formed mesenchymal cells to then form the endoderm, they must undergo an additional process called mesenchymal to epithelial transition (MET). MET is a reverse of EMT where mesenchymal cells convert once again to a fixed epithelial phenotype (Figure 1) [29]. In adult tissues, EMT also plays a role in organ fibrosis and wound healing. Recent studies demonstrated that EMT played a role in development of fibrosis as epithelial cells were able to migrate and aggregate in other locations [30]. Thus, suppression of EMT can potentially be utilized as novel therapy for patients with organ fibrosis.

## 3. Cripto-1: Role in EMT and Cancer Stem Cells (CSC)

Cripto-1 proved to play a pivotal role in the epithelial to mesenchymal transition process as it moderates cell movement and EMT in embryogenesis [7,9]. In gastrulation, Cripto-1 operates primarily at the beginning during the primitive streak phase and spreads to the embryonic mesoderm. It is reduced in the early neural lateral plate stage and disappears in the late neural plate stage [6,31]. Therefore, Cripto-1 expression declines within gastrulation until it disappears and cannot be detected in later parts of embryogenesis under normal physiological conditions. 

While EMT is a useful process to mobilize and further differentiate cells within embryogenesis, it can also be involved with various disease etiologies, particularly cancer. Cells that undergo EMT can become more mobile and move throughout the body to a new, inappropriate location. One mechanism by which this can happen is through a homozygous loss-of-function mutation of E-cadherin. This complete loss of function causes the epithelia to form incorrectly, and epithelial cells fail to adhere together, which is fatal for blastocyst development [32]. Loss of heterozygosity toward the homozygous mutation of CDH1, the gene that encodes E-cadherin, is involved in cancer metastasis, particularly for breast, liver, and prostate cancers [33]. At the new location, they can then convert back to the epithelial phenotype via MET (Figure 1) [29]. Thus, malignant cells can migrate to other areas of the body and implant themselves inappropriately. Carcinomas frequently use EMT to metastasize. One example of this is CS-99 human carcinosarcoma, which requires both EMT and MET to metastasize to the lungs [34]. Some studies have found that some cancers that initially undergo EMT will use an MET-independent pathway to still metastasize and implant in a different epithelium [34,35]. These metastasizing cancers, however, all use EMT to initially metastasize, and Cripto-1 operates within the epithelial to mesenchymal transition process, but not in the mesenchymal to epithelial process. 

Within EMT, cells that need to cluster at the end of dorso-ventral migration will express N-cadherin to further aggregate. In cancer, an increase in N-cadherin expression contributes to the mobilization and aggregation of metastatic cancer cells through a variety of mechanisms. Studies have demonstrated that breast, prostate, bladder, thyroid, and oral squamous cell carcinomas exhibit de novo synthesis of N-cadherin, while melanoma tends to have a re-expression of N-cadherin that was previously expressed in the melanocyte precursors. On the other hand, pleural mesothelioma undergoes an upregulation of a pre-existing baseline level of N-cadherin expression. N-cadherin is found to be downregulated only in few types of cancer, such as osteosarcoma, which leads to inhibition of cell migration and metastasis [24]. As previously mentioned, Cripto-1 downregulates E-cadherin and upregulates N-cadherin (Figure 2). While Cripto-1 and its co-receptor Nodal, another important embryonic protein in the TGF-β signaling pathways [5], have increased expression in various cancers, the two proteins promote EMT through mechanisms independent of one another. It has been established that Nodal induces EMT through phosphorylation of ERK1/2 [36] while Cripto-1 uses c-SRC signaling to induce EMT [7]. However, this article only focuses on the involvement of Cripto-1 in EMT in different types of cancer.

Importantly, Cripto-1 also plays a crucial role in the development and maintenance of cancer stem cells (CSC) [37,38]. Cancer stem cells are known to cause tumor relapse and drug resistance as they promote tumor growth and metastasis [39,40,41,42]. Cripto-1 was found to be highly expressed in stem cell-like human malignant melanomas [37] and in human prostate cell lines [43]. CSCs are notoriously difficult to eliminate due to their resistance to chemotherapeutic agents and radiation therapy. Thus, Cripto-1 could be an ideal target for future therapies to reduce EMT and the generation of cancer stem cells. 

## 4. Cripto-1: Involvement in Different Types of Cancer

### 4.1. Breast Cancer

Cripto-1 is overexpressed in a majority of primary human carcinomas including breast cancers. Cripto-1 induces in vitro cell transformation, migration, invasion, epithelial to mesenchymal transition (EMT), and branching morphogenesis in human breast tissues [8,11,44,45,46,47,48]. Moreover, Cripto-1 can be characterized as an oncogene based on studies that demonstrated Cripto-1 transgene’s ability to form mammary hyperplastic lesions and adenocarcinoma of the mammary gland in multiparous transgenic mice that express human Cripto-1 under the murine mammary tumor virus (MMTV) promoter [45]. This transgenic mouse model constantly expresses increased levels of dephosphorylated (active) β-catenin and phosphorylated (inactive) GSK-3β. Importantly, the mammary tumors from the MMTV/Cripto-1 transgenic mice display features of epithelial to mesenchymal transition, including reduced expression of the epithelial marker E-cadherin and increased expression of the mesenchymal markers, Vimentin and N-cadherin, compared to the normal counterpart FVB multiparous mice [7].

Intriguingly, an inverse correlation between the expression of Snail, a known trigger of epithelial to mesenchymal transition, and the prognosis of breast cancers has been documented [49]. Studies revealed that Snail expression correlates directly with the differentiation status and the invasive and metastatic abilities of mouse and human breast cancer cell lines and tissues [50]. 

Similarly, the developmental transcription factor Msx2 has been shown to induce EMT by up-regulating Cripto-1 expression in the immortalized normal mouse mammary epithelial cells, NMUMG, as evident in the accompanied reduced expression of E-cadherin and increased expression of the mesenchymal phenotype proteins, Vimentin and N-cadherin. Moreover, this mesenchymal phenotype of Msx2-expressing cells can be reversed into a more epithelial-like phenotype by inhibiting Cripto-1 signaling using specific anti-Cripto-1 small-interfering RNA or blocking the c-Src signaling pathway [8]. Altogether, these findings suggest that Msx2 and Cripto-1 collaborate in promoting EMT and contribute to breast tumor progression and invasion processes. 

Moreover, Cripto-1 indirectly induces the phosphorylation of Erb-B2 receptor tyrosine kinase 4 (erbB4), which seems to be a necessary step in the Cripto-1-mediated activation of the mitogen-activated protein kinase (MAPK) signaling pathway [51].

Cripto-1 also promotes tumor angiogenesis, an important hallmark of cancer. It has been established that Cripto-1 significantly increases the proliferation and migration of human umbilical vein endothelial cells (HUVECs) and stimulates new blood vessels’ formation in mouse models of breast cancer. Cripto-1 angiogenic effects seem to be independent of those of the vascular endothelial growth factor (VEGF) [46].

Additionally, Cripto-1 promoter is shown to be highly methylated in most breast cancer cell lines studied to date, including T47D and MCF-7 cells. Treatment with histone deacetylase inhibitors (HDAC) and a demethylating agent can reactivate Cripto-1 expression and results in an aggressive phenotype characterized by enhanced migratory and invasive abilities in the human mammary gland adenocarcinoma MCF-7 cell line [47]. While histone deacetylase inhibitors can inadvertently activate other genes responsible for the aggressive phenotype, co-treatment with anti-Cripto-1 small interfering RNA seems to abrogate the HDAC-driven enhanced migratory abilities of MCF7 cells, suggesting a role for Cripto-1 in the migratory and invasive behavior of breast cancers. 

Finally, the resistance to anoikis, a form of apoptosis induced when the interaction between epithelial cells and extracellular matrix is disrupted, is enhanced in MCF-7 human breast cancer cells with the overexpression of Cripto-1 due to the activation of Akt [11]. Future studies might shed more light on Cripto-1 involvement in breast cancer progression as the scientific evidence currently remains inconclusive.

### 4.2. Lung Cancer

In patients with Stage I non-small cell lung cancer (NSCLC), high Cripto-1 expression correlates with metastatic disease and worse prognosis as shown by postoperative survival curves [52]. Although, detection of Cripto-1 using commercially-available antibodies may be subject to reconsideration due to the lack of specificity of some of these antibodies [53]. Importantly, overexpression of Cripto-1 can be detected in about half of the biopsies from lung adenocarcinoma (LAC) patients where it correlates with several clinical and pathological parameters, including tumor size, lymph involvement, advanced TNM clinical stages [54], and loss of E-cadherin expression. Moreover, patients who are intrinsically resistant to epidermal growth factor receptor-tyrosine kinase inhibitors (EGFR-TKI) treatment are more likely to show overexpression of Cripto-1. Patients in clinical stages I, II, and III with high Cripto-1 have a significantly lower 5-year progression-free survival (PFS) rate and median survival time than those with low Cripto-1 expression. When Cripto-1 expression is combined with carcinoembryonic antigen (CEA) levels higher than 5 ng/mL, the cancer is more progressive, and prognosis is typically poor. Altogether, the combination of Cripto-1 expression and CEA levels might be a useful prognostic indicator to predict tumor progression and survival in lung adenocarcinoma patients [10].

### 4.3. Gastrointestinal Tract Cancers

In a recent study, Cripto-1 and its binding partner, Nodal, were found to be re-expressed in oral squamous cell carcinoma (OSCC) tissue samples. Additionally, their downstream signaling molecules Src, which is associated with the function of Cripto-1, and ERK1/2, which is involved in Nodal signaling, were detected in their phosphorylated active form in the invasive squamous cell carcinoma cell lines SCC15 and SCC25. Additionally, this study attempted to simultaneously block Nodal and Cripto-1 signaling in SCC15 and SCC25 using a blocking antibody, WS65 and a bicyclic peptide, B3, made to prevent Cripto-1 from binding to ALK4 receptor. This blockade of both Nodal and Cripto-1 signaling pathways resulted in significant reduction in the viability of the treated cells, which was accompanied by reduced activation of the signaling molecules, P-Src and P-ERK1/2, associated with Cripto-1 and Nodal, respectively. Moreover, the combined treatment seemed to diminish the aggressiveness of SCC25 cells as measured by a transwell migration and invasion assay [55].

Cripto-1 might also serve as an independent prognostic biomarker in esophageal squamous cell carcinoma (ESCC) since Cripto-1 expression levels positively correlates with the depth of invasion, lymph node involvement, late TNM stages [54], and poor overall survival. Moreover, Cripto-1 could be a marker for ESCC cancer stem-like cells (ECSLC) since ESCC cells with high levels of Cripto-1 possess cancer stem-like (CSL) properties. Indeed, the flow cytometry-sorted Cripto-1^high^ subpopulations of the EC109 and TE-1 human esophageal squamous cell carcinoma cell lines exhibit higher levels of the stemness-related transcription factors Sox2, Oct4 and Nanog, and display a stronger capacity of sphere and colony formation in vitro as well as higher tumorigenicity and metastatic potential in a xenograft nude mouse model. Additionally, Cripto-1^high^ cells exhibit EMT-related gene expression pattern including reduced expression of E-cadherin and increased expression of Vimentin, Snail, E47, Foxc2, SIX1, ZEB1, ZEB2 and MMP9. The Cripto-1-induced EMT is associated with enhanced invasive and metastatic abilities of the Cripto-1^high^ esophageal squamous cell carcinoma cells. Consequently, silencing of Cripto-1 expression by RNA interference reduces the gene expression of factors related to stemness and epithelial to mesenchymal transition [38].

Immunohistochemistry studies have documented an upregulated Cripto-1 expression and downregulated E-cadherin expression in primary gastric carcinoma compared to the counterpart normal gastric mucosa. These studies established a strong correlation between gastric malignancies with Cripto-1 expression with no E-cadherin expression (CR^+^/E-cad^−^) and several aggressive clinicopathological features, including positive lymph node metastasis, advanced TNM stages [54], and the presence of liver metastases. Thus, a combined Cripto-1 and E-cadherin expression analysis can potentially predict the metastatic behavior and determine the overall prognosis for patients with gastric adenocarcinoma [56].

Patients with colon carcinomas show significantly higher serum Cripto-1 levels than healthy individuals [57]. Immunohistochemical analysis demonstrated that Cripto-1 is abundant in colorectal cancer samples and that its expression is positively correlated with tumor size, depth of invasion, lymph node involvement, liver metastasis, and advanced TNM stages [54]. Moreover, the expression of Cripto-1 correlates with worse overall survival (OS) in all clinical stages and with decreased disease-free survival in patients with stage 0-III colorectal cancer. Cripto-1 is postulated to play a crucial role in the carcinogenesis, progression and metastasis in colorectal cancer since Akt and MAPK signaling pathways are activated by Cripto-1 and the knockdown of Cripto-1 can inhibit proliferation and migration in colorectal cancer cell lines [58].

### 4.4. Liver Cancer

Patients with hepatocellular carcinoma (HCC) typically have elevated serum Cripto-1 levels, especially those with HBV-related HCC and cirrhosis [48]. A positive correlation between serum Cripto-1 levels and the α-feto-protein (AFP) has been documented in HBV-related HCC patients. Similarly, larger tumors (<5 cm in diameter), lymph node involvement, distant metastases advanced TNM [54] and Barcelona clinic liver cancer (BCLC) stages, as well as tumor recurrences positively correlated with high levels of Cripto-1 in HCC. It was found that overexpression of Cripto-1, high AFP level, high gamma-glutamyl transferase (GGT) level, liver cirrhosis, larger tumors, and vascular invasion lead to poor overall survival (OS) in HCC patients. Similarly, shorter time to recurrence (TTR) significantly correlates with overexpression of Cripto-1, high AFP level, high GGT level, liver cirrhosis, larger tumor size, satellite nodule, and vascular invasion. On the other hand, HCC patients with low Cripto-1 expression have significant OS and TTR advantages. Therefore, Cripto-1 proved to be a reliable predictor for OS and TTR in HCC patients, the 5-year OS and TTR rates in those patients with high Cripto-1 are 37% and 23% compared to OS of 66% and TTR of 62% for patients with low Cripto-1. Similarly, Cripto-1 levels are significantly higher in advanced TNM and BCLC stages. Additionally, patients with overexpression of Cripto-1 are almost three times more likely to relapse. Altogether, this suggests that Cripto-1 can be utilized as an independent prognostic biomarker, or in combination with AFP values, for the different groups of HCC patients. Moreover, there is a close association between capsular infiltration and matrix metallopeptidase 9 (MMP-9) levels in HCC tumors. Interestingly, patients with high MMP-9 levels have high Cripto-1 levels as well [59].

### 4.5. Renal Cancer

Cripto-1 mRNA and Cripto-1 protein were found to be significantly overexpressed in clear cell renal cell carcinoma (ccRCC) tissue samples compared to matched adjacent normal tissues. T status, lymph-node status, distant metastasis, TNM stage and Fuhrman grade are all positively associated with Cripto-1 expression. Overall survival (OS) and recurrence free survival (RFS) are significantly shorter in patients with overexpression of Cripto-1. Additionally, serum Cripto-1 levels are higher in ccRCC patients and its values positively correlate with tumor size, Fuhrman grade, and TNM stage [54]. Moreover, a decrease in serum Cripto-1 levels four weeks post-operation compared to pre-operation is likely to reflect tumor burden, while another post-operation increase in Cripto-1 levels can reflect recurrence. Both the in vitro and in vivo proliferation and tumorigenesis of ccRCC seem to be promoted by Cripto-1 since silencing of Cripto-1 by RNA interference inhibits tumor growth in a nude mouse xenograft model. Studies have shown that overexpression of Cripto-1 enhances ccRCC cell migration, angiogenesis and invasion abilities, while the knockdown of Cripto-1 expression inhibits migration, reduces angiogenesis, and reduces the frequency of metastasis in vivo. 

Additionally, Cripto-1 promotes EMT in ccRCC cells by activating the Wnt/β-catenin signaling pathway both in vitro and in vivo. Indeed, overexpression of Cripto-1 decreased E-cadherin expression and increased the expression of Vimentin, N-cadherin, β-catenin, ZEB-1, and Snail, as well as the matrix metalloproteinases (MMP-2 and MMP-9) that are involved in tumor invasion and metastasis due to their role in degrading the extracellular matrix [60]. 

### 4.6. Reproductive System Cancers

In vitro studies have shown that transfection of the metastatic cervical epidermoid carcinoma Ca Ski cells [61] results in a six-fold increase in the mesenchymal intermediate filament protein Vimentin, which is associated with a more invasive phenotype in cervical carcinomas. Cripto-1-transfected Ca Ski cells also display a significant increase in their abilities to migrate through collagen- or gelatin-coated membranes in a dose-dependent manner. Administration of exogenous recombinant Cripto-1 also yielded similar outcome [12]. 

Similarly, Cripto-1 treatment of SiHa, a cervical squamous cell carcinoma cell line that contains an integrated human papillomavirus type 16 genome (HPV-16) [62,63], leads to a significant increase in tyrosine phosphorylation of the p85 regulatory subunit of PI3K while treatment with LY294002, a potent inhibitor of PI3Ks, abrogates the p85 phosphorylation. Interestingly, treatment of SiHa and Ca Ski cervical carcinoma cells with Cripto-1 induces hyperphosphorylation of AKT leading to anti-apoptotic effects suggesting a role for Cripto-1 as a survival factor in cervical malignancies through a PI3K/Akt/GSK-3β pathway [64]. 

In primary ovarian cancers, Cripto-1 immunoreactivity can be detected in a significant portion of tissue samples, especially in the serous and mucinous histological types of ovarian carcinoma. Cripto-1 is usually found co-expressed with amphiregulin (AR), and transforming growth factor α (TGFα) in a majority of ovarian carcinoma samples. However, only AR expression correlates with the clinical parameter of histopathological grade that represents the degree of differentiation in cancer cells, where AR is detected more frequently in the low grade 1 and 2 tumors than in the high grade 3 ovarian tumors [65]. 

Studies have shown that prostate cancer patients with overexpression of Cripto-1 have a poor overall prognosis following radical prostatectomy. Approximately 42% of prostate cancer tissue samples show elevated Cripto-1 and that is significantly higher than in benign prostate hyperplasia (BPH). Cripto-1 expression is increased in the human adenocarcinoma prostate cell lines PC-3 and LNCaP than in normal prostate epithelial cells RWPE-1. The pre-operative prostate-specific antigen (PSA) level, Gleason score, and lymph node metastasis positively correlate with Cripto-1 expression. Biochemical recurrence (BCR)-free survival times varied for those with high and low Cripto-1 expression where Cripto-1 overexpression seems to be associated with shorter BCR-free survival [66]. Importantly, Cripto-1 overexpression correlated with epithelial to mesenchymal transition in prostate cancers. At the cellular level, there is a positive correlation between Cripto-1 expression and β-catenin, a proto-oncogene and a key player in EMT. It has been established that EMT in prostate cancer is regulated by Cripto-1 via the Wnt/β-catenin pathway. As a proof of concept, Cripto-1 can be targeted by RNA interference. In recent studies, Cripto-1 expression was silenced in PC-3 cells upon transfection with short hairpin RNA (shRNA). Silencing of Cripto-1 helps reverse EMT as indicated by increased expression of E-cadherin, which seems to be dependent on the Wnt/β-catenin pathway in the PC-3 cell line. Moreover, Cripto-1 silencing suppresses prostate cancer cell invasion and migration abilities [9].

### 4.7. Cutaneous Melanoma

Studies have shown that about half of examined melanoma tissue biopsies and melanoma cell lines express Cripto-1 to a varying extent. Moreover, Cripto-1 is involved in the growth, proliferation and invasion of these malignant cells. Treatment of melanoma cell lines with recombinant Cripto-1 activates c-Src as evident in the significant increase in c-Src phosphorylation. Additionally, exogenous Cripto-1 enhances the invasiveness of the treated melanoma cell lines. Conversely, co-treatment with Saracatinib, a specific c-Src inhibitor, abrogates the c-Src phosphorylation and reduces the invasive abilities of melanoma cell lines. The inhibitory effects of Saracatinib correspond to the levels of endogenous Cripto-1 expression in the different melanoma cell lines. Interestingly, silencing of endogenous Cripto-1 expression using a pool of anti-Cripto-1 small interfering RNA (siRNA) significantly reduces the growth of melanoma cells and inhibits their invasiveness [67]. 

Human melanoma cells that express cell surface Cripto-1 can be isolated using fluorescence-activated cell sorting (FACS). The resulting subpopulation of melanoma cells possesses stem cell-like characteristics including the expression of Oct4, MDR-1 and activated c-Src. These stem-like cells might be responsible for the progression and recurrence of malignant melanomas since the progeny of these cells is characterized by increased growth rate and aggressiveness following subsequent transplants in immunocompromised mice [37].

## 5. Cripto-1 as a Therapeutic Target in Cancer

Given Cripto-1′s role in cancer, especially in cancer stem cells, it is an ideal target for precise therapies, such as blocking monoclonal antibodies, especially since it is detected in higher levels in cancers compared to healthy tissues [68]. In general, anti-Cripto-1 blocking antibodies have shown promising therapeutic effects. For instance, blockade of both Cripto-1 and Nodal signaling is shown to inhibit cell viability and invasiveness in oral squamous cell carcinoma cells [55]. Several anti-Cripto-1 antibodies have been developed, including the antibody-cytotoxic conjugate, BIIB015 [69]. Despite the encouraging preclinical data, the phase I-clinical trial (NCT00674947) to evaluate the safety and maximum-tolerated dose (MTD) of BIIB015 ended in 2011 without the publication of its clinical outcome. Other immunotherapeutic agents include a monoclonal antibody that disrupts AKT signaling [70] and a novel humanized Cripto-1 antibody [71]. Moreover, anti-Cripto-1 monoclonal antibodies can target tumor angiogenesis since they are shown to inhibit microvessel formation in vivo [46]. Intriguingly, Cripto-1 has also been utilized as an oncofetal tumor antigen in vaccination immunotherapies resulting in robust cytotoxic T cell-mediated immune responses against cancer stem cells and metastases in melanoma and breast cancer animal models [72,73]. Additionally, it has been documented that treatment with antisense oligonucleotides directed against Cripto-1 results in a significant inhibition of the growth of human cancer cells of different histological origins, where Cripto-1 functions as either an autocrine or paracrine growth factor [11,74,75,76]. Altogether, Cripto-1 might serve as a promising target for effective treatments of different types of cancer and to prevent metastatic disease and relapse, especially through specific targeting of small molecules, small interfering RNA, and monoclonal antibodies.
